# Supplementary material for: Changes in Spo0A~P pulsing frequency control biofilm matrix deactivation
Source: PLoS Comput Biol. 2025 Jul 7;21(7):e1013263. doi: 10.1371/journal.pcbi.1013263 (PMC12270306; doi:10.1371/journal.pcbi.1013263)
Supplement: S1 Text — Supplementary tables A-F and supplementary figures A-E. (PDF) [file pcbi.1013263.s001.pdf]

SUPPLEMENTAL MATERIAL FOR

Changes in Spo0A~P pulsing frequency control biofilm matrix deactivation

Cristina S. D. Palma[1], Daniel J. Haller[2], Jeffrey J. Tabor[1,2,3,4,5], Oleg A. Igoshin[1,3,5,6,7]*

[1]Department of Bioengineering, Rice University, Houston, Texas, United States of America
[2]Systems, Synthetic, and Physical Biology Ph.D. Program, Rice University, Houston, Texas, United States of America
[3]Department of Biosciences, Rice University, Houston, Texas, United States of America
[4]Department of Chemical and Biomolecular Engineering, Rice University, Houston, Texas, United States of America
[5]Rice Synthetic Biology Institute, Rice University, Houston, Texas, United States of America
[6]Center for Theoretical Biological Physics, Rice University, Houston, Texas, United States of America
[7]Department of Chemistry, Rice University, Houston, Texas, United States of America

* igoshin@rice.edu

This PDF includes:

Supplementary Tables: S2
Supplementary Figures: S8
Supplementary References: S13

Supplementary Tables:

Table A: Kinetic parameters and model reactions of the phosphorelay model (Methods section 4.1) and (1). Subscript *P* marks the protein phosphorylated form. The '_' symbol denotes protein complexes. For all proteins the degradation rate was fixed at 0.3 hr^{-1} (1). In the table, *g* stands for 'growth rate', *n* for the copy number of the gene, and *F(g)* is the function describing how the cell volume changes with the growth rate (Methods Section 4.1). As is (1), in R1 to R4, we assume the existence of a delay between the change in protein production initiation rate (v_p^i) and the protein production rate at the current time (v_p^c), where p = {kinA; 0F; 0B; 0A} (Methods section 4.1). Due to space constraints, 0A~P was abbreviated as $0A_P$.

#	Event	Reaction	Rate constants
**R1**	Production of Kinase A	$\xrightarrow{n \cdot v_{kinA}^c} \text{KinA}$	$v_{kinA}^i = \left(v_{kinA}^0 + v_{kinA}^{\max} \cdot \frac{[0A_P]^m}{K_{kinA}^m + [0A_P]^m} \right) \cdot [F(g)]$ $v_{kinA}^0 = 0.9$ µM.h^{-1}; $v_{kinA}^{\max} = 1.5$ µM.h^{-1}; $K_{kinA} = 0.025$ µM; $m = 1$
**R2**	Production of phospho-transferase Spo0F	$\xrightarrow{n \cdot v_{0F}^c} 0\text{F}$	$v_{0F}^i = \left(v_{0F}^0 + v_{0F}^{\max} \cdot \frac{[0A_P]^m}{K_{0F}^m + [0A_P]^m} \right) \cdot [F(g)]$ $v_{0F}^0 = 0.15$ µM.h^{-1}; $v_{0F}^{\max} = 3$ µM.h^{-1}; $K_{0F} = 0.15$ µM; $m = 2$
**R3**	Production of Spo0B	$\xrightarrow{n \cdot v_{0B}^c} 0\text{B}$	$v_{0B}^i = v \cdot [F(g)]$; $v = 0.3$ µM.h^{-1}
**R4**	Production of Spo0A	$\xrightarrow{n \cdot v_{0A}^c} 0\text{A}$	$v_{0A}^i = \left(v_{0A}^0 + v_{0A}^{\max} \cdot \frac{[0A_P]^m}{K_{0A}^m + [0A_P]^m} \right) \cdot [F(g)]$ $v_{0A}^0 = 1.5$ µM.h^{-1}; $v_{0A}^{\max} = 6$ µM.h^{-1}; $K_{0A} = 0.35$ µM; $m = 2$
**R5**	Production of phosphatase Rap	$\xrightarrow{n \cdot v_{Rap}} Rap$	$v_{Rap} = v_1 \cdot [F(g)]$; $v_1 = 0.075$ µM.h^{-1}
**R6**	Production of phosphatase Spo0E	$\xrightarrow{n \cdot v_{0E}} 0\text{E}$	$v_{0E} = v_2 \cdot [F(g)]$; $v_2 = 0.03$ µM.h^{-1}
**R7**	KinA auto phosphorylation	$\text{KinA} \underset{k_{dp}}{\overset{k_p}{\rightleftarrows}} \text{KinA}_P$	$k_p = 12$ h^{-1}; $k_{dp} = 1$ h^{-1}
**R8**	0F phosphorylation	$\text{KinA}_P + 0\text{F} \underset{k_1}{\overset{k_b}{\rightleftarrows}} \text{KinA}_P\text{\_0F} \underset{k_b}{\overset{k_2}{\rightleftarrows}} \text{KinA} + 0\text{F}_P$	$k_b = 5$ nM^{-1}h^{-1}; $k_1 = 500$ h^{-1}; $k_2 = 300$ h^{-1}
**R9**	Inhibition of KinA phosphorylation due to complex formation with 0F	$\text{KinA} + 0\text{F} \underset{k_i}{\overset{k_b}{\rightleftarrows}} \text{KinA\_0F}$	$k_b = 5$ nM^{-1}h^{-1}; $k_i = 500$ h^{-1};
**R10**	0F to 0B phosphotransfer	$0\text{F}_P + 0\text{B} \underset{k_3}{\overset{k_b}{\rightleftarrows}} 0\text{F}_P\text{\_0B} \underset{k_b}{\overset{k_4}{\rightleftarrows}} 0\text{F} + 0\text{B}_P$	$k_b = 5$ nM^{-1}h^{-1}; $k_3 = 200$ h^{-1}; $k_4 = 800$ h^{-1}
**R11**	0B to 0A phosphotransfer	$0\text{B}_P + 0\text{A} \underset{k_5}{\overset{k_b}{\rightleftarrows}} 0\text{B}_P\text{\_0A} \underset{k_b}{\overset{k_6}{\rightleftarrows}} 0\text{B} + 0\text{A}_P$	$k_b = 5$ nM^{-1}h^{-1}; $k_5 = 200$ h^{-1}; $k_6 = 800$ h^{-1}
**R12**	$0F_P$ dephosphorylation	$0\text{F}_P + \text{Rap} \underset{k_{ubr}}{\overset{k_b}{\rightleftarrows}} 0\text{F}_P\text{\_Rap} \xrightarrow{k_r} 0\text{F} + \text{Rap}$	$k_b = 5$ nM^{-1}h^{-1}; $k_{ubr} = 100$ h^{-1}; $k_r = 100$ h^{-1}
**R13**	$0A_P$ dephosphorylation	$0\text{A}_P + 0\text{E} \underset{k_{ube}}{\overset{k_b}{\rightleftarrows}} 0\text{A}_P\text{\_0E} \xrightarrow{k_e} 0\text{A} + 0\text{E}$	$k_b = 5$ nM^{-1}h^{-1}; $k_{ube} = 100$ h^{-1}; $k_e = 100$ h^{-1}

Table B: Model differential equations of the phosphorelay model (1). The rate constant values are shown in Table A. Subscript '*tot'* stands for 'total concentration'. In the table, *n* stands for the copy number of the gene. Unphosphorylated species are calculated from conservation laws (*e.g.* $[KinA]=[KinA_{tot}]-[KinA_p]-[KinA_p\_0F]-[KinA\_0F]$). As is (1), in E1 to E4, we assume the existence of a delay between the change in protein production initiation rate v_p^i and the protein production rate at the current time v_p^c**,** where *p* = {kinA; 0F; 0B; 0A} (Methods section 4.1). Due to space constraints, 0A~P was abbreviated as $0A_P$.

#	Model differential equations
**E1**	$\frac{d[KinA_{tot}]}{dt}=n\cdot v_{kinA}^c\text{ -}k_{deg\_P}\cdot[KinA_{tot}]$
**E2**	$\frac{d[0F_{tot}]}{dt}=n\cdot v_{0F}^c\text{-}k_{deg\_P}\cdot[0F_{tot}]$
**E3**	$\frac{d[0B_{tot}]}{dt}=n\cdot v_{0B}^c\text{ -}k_{deg\_P}\cdot[0B_{tot}]$
**E4**	$\frac{d[0A_{tot}]}{dt}=n\cdot v_{0A}^c\text{ -}k_{deg\_P}\cdot[0A_{tot}]$
**E5**	$\frac{d[Rap_{tot}]}{dt}=n\cdot v_{Rap}\text{ -}k_{deg\_P}\cdot[Rap_{tot}]$
**E6**	$\frac{d[0E_{tot}]}{dt}=n\cdot v_{0E}\text{-}k_{deg\_P}\cdot[0E_{tot}]$
**E7**	$\frac{d[KinA_p]}{dt}=k_p\cdot[KinA]\text{-}k_{dp}\cdot[KinAp]\text{-}k_b\cdot[KinAp]\cdot[0F]+k_1\cdot[KinA_p\_0F]\text{-}k_{deg\_P}\cdot[KinA_p]$
**E8**	$\frac{d[KinA_p\_0F]}{dt}=k_b\cdot[KinA_p]\cdot[0F]\text{-}(k_1+k_2+k_{deg\_P})\cdot[KinA_p\_0F]+k_b\cdot[KinA]\cdot[0F_p]$
**E9**	$\frac{d[0F_p]}{dt}=k_2\cdot[KinA_p\_0F]\text{-}k_{deg\_P}\cdot[0F_p]\text{-}k_b\cdot[0F_p]\cdot[0B]+k_3\cdot[0F_p\_0B]\text{-}k_b\cdot[0F_p]\cdot[Rap]+k_{ubr}\cdot[0F_p\_Rap]\text{-}k_b\cdot[KinA]\cdot[0F_p]$
**E10**	$\frac{d[KinA\_0F]}{dt}=k_b\cdot[KinA]\cdot[0F]\text{-}(k_i+k_{deg\_P})\cdot[KinA\_0F]$
**E11**	$\frac{d[0B_p]}{dt}=\text{-}k_{deg\_P}\cdot[0B_p]\text{-}k_b\cdot[0F]\cdot[0B_p]+k_4\cdot[0F_p\_0B]\text{-}kb\cdot[0A]\cdot[0B_p]+k_5\cdot[0B_p\_0A]$
**E12**	$\frac{d[0F_p\_0B]}{dt}=k_b\cdot([0F_p]\cdot[0B]+[0B_p]\cdot[0F])\text{-}[0F_p\_0B]\cdot(k_{deg\_P}+k_3+k_4)$
**E13**	$\frac{d[0A_p]}{dt}=\text{-}k_{deg\_P}\cdot[0A_p]\text{-}k_b\cdot[0B]\cdot[0A_p]+k_6\cdot[0B_p\_0A]\text{-}k_b\cdot[0A_p]\cdot[0E]+k_{ube}\cdot[0A_p\_0E]$
**E14**	$\frac{d[0B_p\_0A]}{dt}=k_b\cdot([0A_p]\cdot[0B]+[0B_p]\cdot[0A])\text{-}[0B_p\_0A]\cdot(k_{deg\_P}+k_5+k_6)$
**E15**	$\frac{d[0F_p\_Rap]}{dt}=k_b\cdot[0F_p]\cdot[Rap]\text{-}(k_{ubr}+k_r+k_{deg\_P})\cdot[0F_p\_Rap];$
**E16**	$\frac{d[0A_p\_0E]}{dt}=k_b\cdot[0A_p]\cdot[0E]\text{-}(k_{ube}+k_e+k_{deg\_P})\cdot[0Ap\_0E];$

Table C: Kinetic parameters and model reactions of the biofilm matrix production deterministic model. The rate constants were converted from the model in (2), assuming a cell volume of 4fL (3). In the table, *g* stands for 'growth rate', *n* for the copy number of the gene, and *F(g)* is the function describing how the cell volume changes with the growth rate (Methods Section 4.2). The degradation rate of RNA $\left(k_{deg}^{m}\right)$ was set to 8.3 h^{-1} (4). The degradation rate of all proteins $\left(k_{deg}^{pro}\right)$ was set to 0.2 h^{-1} (5), except for SlrR which was set to 0.8 h^{-1}, given that it is known to be an unstable protein (6). The relative transcription, translation and K_i rates were set to ensure the bifurcation diagram resulted in the transitions from matrix OFF state to matrix ON state at a realistic growth rate and 0A~P ($0A_p$) level.

#	Event	Reaction	Rate constants
**R1**	Production of SinI	$\xrightarrow{v_{SinI}} \mathrm{I}$	$v_{SinI} = \left(v_i^0 + v_i^{\max} \cdot \frac{[0A_P]}{K_i + [0A_P]}\right) \cdot \left(n \cdot F(g)\right) \cdot \left(\frac{k_{tran}^{i}}{k_{\deg}^{m}}\right)$ $v_i^0 = 0;\ v_i^{\max} = 0.03\ \mu\mathrm{M.h^{-1}};\ K_i = 0.01\ \mu\mathrm{M};$ $k_{tran}^{i} = 400\ \mathrm{h^{-1}};\ k_{\deg}^{m} = 8.3\ \mathrm{h^{-1}};$
**R2**	Production of SinR	$\xrightarrow{v_{SinR}} \mathrm{R}$	$v_{SinR} = v_r \cdot \left(n \cdot F(g)\right) \cdot \left(\frac{k_{tran}^{r}}{k_{\deg}^{m}}\right)$ $v_R = 0.05\ \mu\mathrm{M.h^{-1}};\ k_{tran}^{r} = 200\ \mathrm{h^{-1}};\ k_{\deg}^{m} = 8.3\ \mathrm{h^{-1}}$
**R3**	Production of SlrR	$\xrightarrow{v_{SlrR}} \mathrm{L}$	$v_{SlrR} = \left(v_l^0 + v_l^{\max} \cdot \frac{K_l}{K_l + [R_T]}\right) \cdot \left(n \cdot F(g)\right) \cdot \left(\frac{k_{tran}^{l}}{k_{\deg}^{m}}\right)$ $v_l^0 = 0;\ v_l^{\max} = 0.046\ \mu\mathrm{M.h^{-1}};\ K_l = 0.9\ \mathrm{nM};$ $k_{tran}^{l} = 200\ \mathrm{h^{-1}};\ k_{\deg}^{m} = 8.3\ \mathrm{h^{-1}};$
**R4**	Production of TapA	$\xrightarrow{v_{TapA}} \mathrm{T}$	$v_{TapA} = \left(v_t^0 + v_t^{\max} \cdot \frac{K_t}{K_t + [R_T]}\right) \cdot \left(n \cdot F(g)\right) \cdot \left(\frac{k_{tran}^{t}}{k_{\deg}^{m}}\right)$ $v_t^0 = 0;\ v_t^{\max} = 0.01\ \mu\mathrm{M.h^{-1}};\ K_t = 2.1\ \mathrm{nM};$ $k_{tran}^{t} = 200\ \mathrm{h^{-1}};\ k_{\deg}^{m} = 8.3\ \mathrm{h^{-1}};$
**R5**	Formation of SinI dimer	$\mathrm{I} + \mathrm{I} \underset{k_{udi}}{\overset{k_{di}}{\rightleftarrows}} \mathrm{I_d}$	$k_{di} = 722\ \mu\mathrm{M^{-1}} \cdot \mathrm{h^{-1}};\ k_{udi} = 169\ \mathrm{h^{-1}};$
**R6**	Formation of SinR tetramer	$\mathrm{R} + \mathrm{R} \underset{k_{udr}}{\overset{k_{dr}}{\rightleftarrows}} \mathrm{R_T}$	$k_{dr} = 722\ \mu\mathrm{M^{-1}} \cdot \mathrm{h^{-1}};\ k_{udr} = 247\ \mathrm{h^{-1}};$
**R7**	Formation of SinI-SinR heterodimer	$\mathrm{I_d} + \mathrm{R} \xrightarrow{k_{b1}} 2\mathrm{IR}$	$k_{b1} = 770\ \mu\mathrm{M^{-1}} \cdot \mathrm{h^{-1}}$
**R8**	Formation of $SlrR_2$-$SinR_2$ heterotetra-mer	$\mathrm{L} + \mathrm{R} \underset{k_{dlr}}{\overset{k_{b2}}{\rightleftarrows}} \mathrm{LR}$	$k_{b2} = 770\ \mu\mathrm{M^{-1}} \cdot \mathrm{h^{-1}};\ k_{dlr} = 0.99\ \mathrm{h^{-1}}$

Table D: Model differential equations of the biofilm matrix production deterministic model (Table C). The rate constant values are shown in Table C.

#	Model differential equations
**E1**	$\frac{d[I]}{dt}=\left(v_i^0+v_i^{\max}\cdot\frac{[0A_P]}{K_i+[0A_P]}\right)\cdot\left(n\cdot F(g)\right)\cdot\left(\frac{k_{tran}^i}{k_{\deg}^m}\right)-k_{di}\cdot[I]\cdot[I]\cdot 2+k_{udi}\cdot[I_d]\cdot 2-k_{\deg\_P}\cdot[I]$
**E2**	$\frac{d[L]}{dt}=\left(v_l^0+v_l^{\max}\cdot\frac{K_l}{K_l+[R_T]}\right)\cdot\left(n\cdot F(g)\right)\cdot\left(\frac{k_{tran}^l}{k_{\deg}^m}\right)-k_{b2}\cdot[L]\cdot[R]+k_{dlr}\cdot[LR]-k_{\deg\_P}^L\cdot[L]$
**E3**	$\frac{d[R]}{dt}=v_r\cdot\left(n\cdot F(g)\right)\cdot\left(\frac{k_{tran}^r}{k_{\deg}^m}\right)-k_{b1}\cdot[I_d]\cdot[R]-k_{dr}\cdot[R]\cdot[R]\cdot 2+k_{udr}\cdot[Rt]\cdot 2+S1$ *where,* $S1=-k_{b2}\cdot[L]\cdot[R]+k_{dlr}\cdot[LR]-k_{\deg\_P}\cdot[R]$
**E4**	$\frac{d[T]}{dt}=\left(v_t^0+v_t^{\max}\cdot\frac{K_t}{K_t+[R_T]}\right)\cdot\left(n\cdot F(g)\right)\cdot\left(\frac{k_{tran}^t}{k_{\deg}^m}\right)-k_{\deg\_P}\cdot[T]$
**E5**	$\frac{d[IR]}{dt}=k_{b1}\cdot[I_d]\cdot[R]\cdot 2-k_{\deg\_P}\cdot[IR]$
**E6**	$\frac{d[Id]}{dt}=-k_{b1}\cdot[I_d]\cdot[R]+k_{di}\cdot[I]\cdot[I]-k_{udi}\cdot[Id]-k_{\deg\_P}\cdot[I_d]$
**E7**	$\frac{d[LR]}{dt}=k_{b2}\cdot[L]\cdot[R]-k_{dlr}\cdot[LR]-k_{\deg\_P}\cdot[LR]$
**E8**	$\frac{d[R_T]}{dt}=k_{dr}\cdot[R]\cdot[R]-k_{udr}\cdot[R_T]-k_{\deg\_P}\cdot[R_T]$

Table E: Kinetic parameters and model reactions of the biofilm matrix production stochastic model (2). All rate constants were set to be the same as in (2). Protein and mRNA degradation reactions are not included in the table.

#	Event	Reaction
**R1**	*sinI* activation by DNA binding of 0A~P	$g_{OFF}^{\sin I} + 0A_P \xrightleftharpoons[k_{OFF}^{i}]{k_{ON}^{i}} g_{ON}^{\sin I}$
**R2**	*slrR* repression by SinR tetramer DNA binding	$g_{ON}^{slrR} + R_T \xrightleftharpoons[k_{ON}^{l}]{k_{OFF}^{l}} g_{OFF}^{slrR}$
**R3**	*tapA* repression by SinR tetramer DNA binding	$g_{ON}^{TapA} + R_T \xrightleftharpoons[k_{ON}^{t}]{k_{OFF}^{t}} g_{OFF}^{TapA}$
**R4**	Transcription of *sinI*	$g_{ON}^{\sin I} \xrightarrow{v_i} \mathrm{i} + g_{ON}^{\sin I}$
**R5**	Transcription of *slr0*	$g_{ON}^{slrR} \xrightarrow{v_r} \mathrm{l} + g_{ON}^{slrR}$
**R6**	Transcription of *tapA*	$g_{ON}^{TapA} \xrightarrow{v_t} \mathrm{t} + g_{ON}^{TapA}$
**R7**	Transcription of *sinR*	$g^{\sin R} \xrightarrow{v_r} \mathrm{r} + g^{\sin R}$
**R8**	Translation of *sinI* mRNA	$i \xrightarrow{k_{tran}^{i}} I + i$
**R9**	Translation of *sinR* mRNA	$r \xrightarrow{k_{tran}^{r}} R + r$
**R10**	Translation of *slrR* mRNA	$l \xrightarrow{k_{tran}^{l}} L + l$
**R11**	Translation of *tapA* mRNA	$t \xrightarrow{k_{tran}^{t}} T + t$
**R12**	Formation of SinI-SinR heterodimer	$\mathrm{I_d} + \mathrm{R} \xrightarrow{k_{b1}} 2\mathrm{IR}$
**R13**	Formation of SinR tetramer	$\mathrm{R} + \mathrm{R} \xrightleftharpoons[k_{udr}]{k_{dr}} \mathrm{R_T}$
**R14**	Formation of SinI dimer	$\mathrm{I} + \mathrm{I} \xrightleftharpoons[k_{udi}]{k_{di}} \mathrm{I_d}$
**R15**	Formation of $SlrR_2$-$SinR_2$ heterotetramer	$\mathrm{L} + \mathrm{R} \xrightleftharpoons[k_{dlr}]{k_{b2}} \mathrm{LR}$

Table F: Extra model reactions added to the stochastic model in Table E to incorporate other sources of transcriptional noise, such as promoter locking due to supercoiling accumulation (7,8). Rate constants were set such that probability of being $g_{OFF}^{\sin I}$ is 6, and the probability of being g_{ON}^{slrR} and g_{ON}^{TapA} is 5. The subscript *sc* stands for 'supercoiled state'.

#	Event	Reaction
**R1**	*sinI* locked due to supercoiling accumulation	$g_{OFF}^{\sin I} \underset{k_U}{\overset{k_L}{\rightleftarrows}} g_{SC}^{\sin I}$
**R2**	*slrR* locked due to supercoiling accumulation	$g_{ON}^{slrR} \underset{k_U}{\overset{k_L}{\rightleftarrows}} g_{SC}^{slrR}$
**R3**	*tapA* locked due to supercoiling accumulation	$g_{ON}^{TapA} \underset{k_U}{\overset{k_L}{\rightleftarrows}} g_{SC}^{TapA}$

Supplementary Figures:

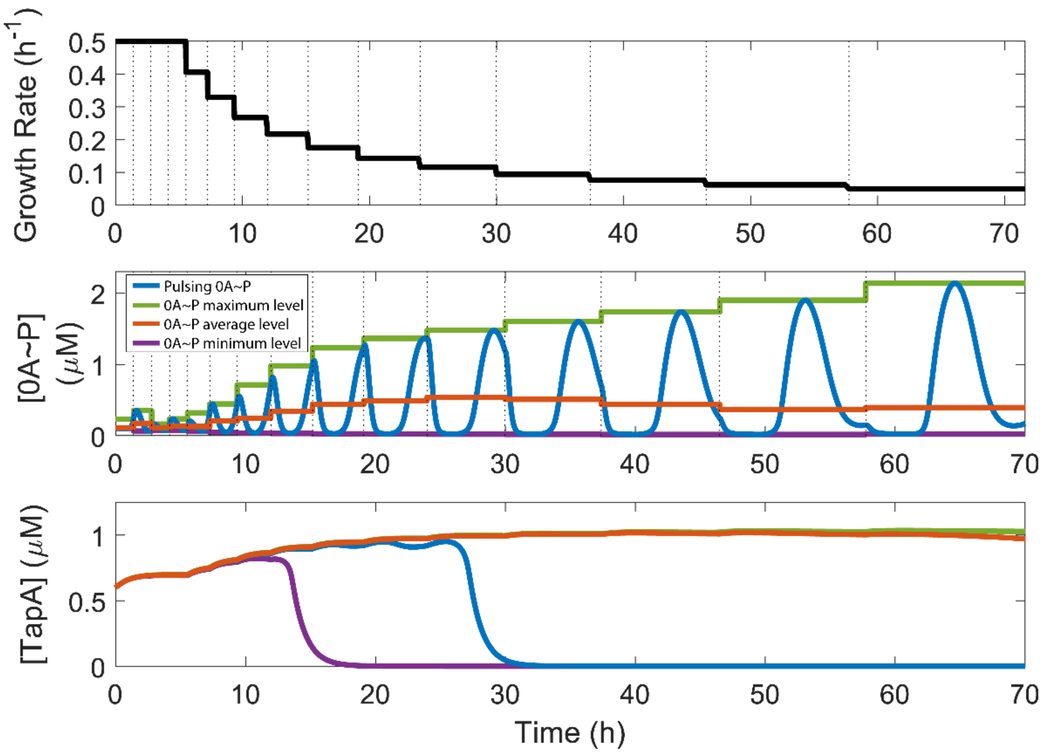

Figure A: Effects of 0A~P dynamics on biofilm matrix deactivation. (Top) Growth rate dynamics used as the input of the phosphorelay and biofilm matrix production mathematical models **(Middle)** 0A~P predicted levels (blue line) from the phosphorelay model assuming the growth rate input dynamics shown in the top plot. Also shown is the 0A~P pulse average level per cell cycle (orange line), the 0A~P pulse maximum level per cell cycle (green line), and the 0A~P pulse minimum level per cell cycle (purple line). Vertical dashed lines represent the beginning of a new cell cycle, according to *Eq. 10*. **(Bottom)** Predicted levels of biofilm matrix protein TapA, estimated from the biofilm matrix production network model. Blue line corresponds to the TapA predicted levels assuming the 0A~P pulsatile input shown in blue color in the middle plot. Orange line corresponds to the TapA predicted levels assuming the average level of 0A~P, per cell cycle, shown by the orange line in the middle plot. Purple line corresponds to the TapA predicted levels assuming the minimum level of 0A~P, per cell cycle, shown by the purple line in the middle plot. Green line corresponds to the TapA predicted levels assuming the maximum level of 0A~P, per cell cycle, shown by the green line in the middle plot.

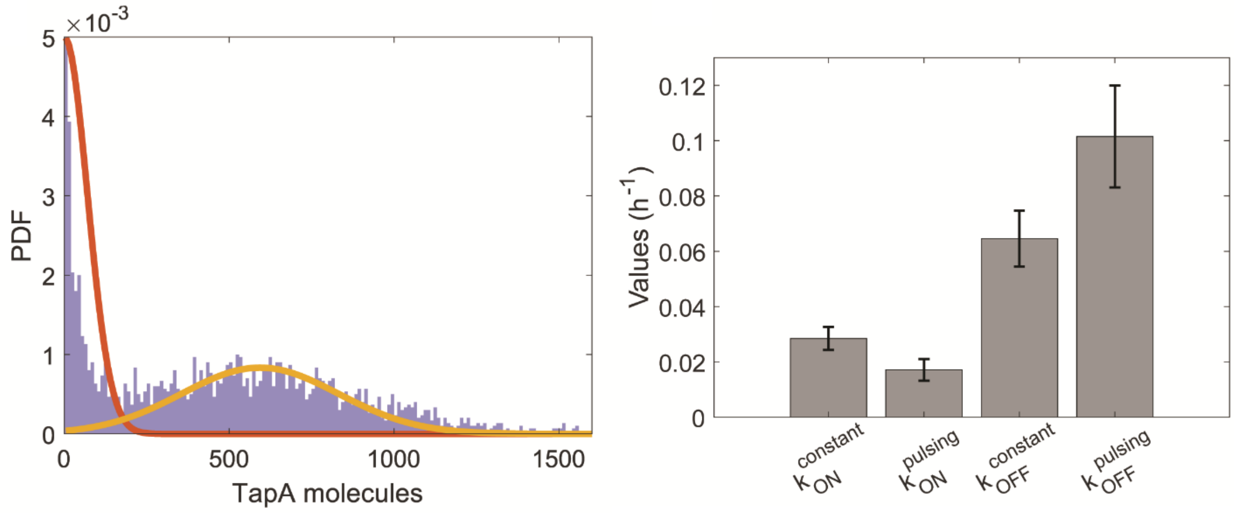

Figure B: (Left) Histogram of TapA molecules obtained from 3000 stochastic simulations (blue bars). Simulation time was set to 50 h. Solid lines represent Gaussian fits derived from a Gaussian Mixture Model with 2 components fitted to the data. The value of 200 molecules was set to be the threshold to distinguish between a biofilm matrix production active and inactive. **(Right)** Mean activation (k_{ON}) and deactivation rates (k_{OFF}) for constant and pulsing 0A~P signals assuming 100 randomly selected thresholds ranging from 25% to 175% of the original 200-molecule value. Vertical error bars correspond to one standard deviation.

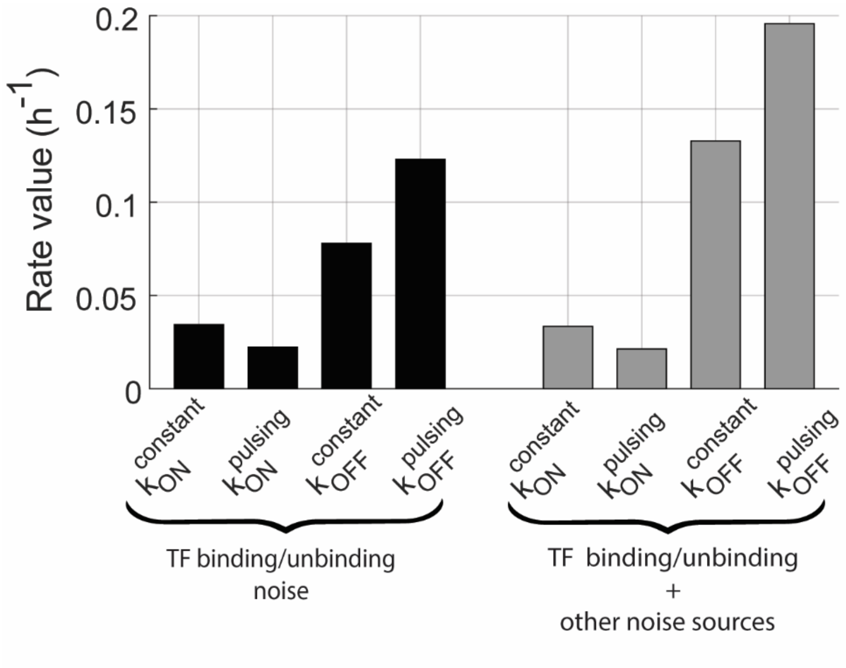

Figure C: Effects of additional transcription bursting. Estimated values of the biofilm matrix activation rate assuming constant ($k_{ON}^{constant}$) and pulsing 0A~P ($k_{ON}^{pulsing}$) and of the matrix deactivation rate assuming constant ($k_{OFF}^{constant}$) and pulsing 0A~P input ($k_{OFF}^{pulsing}$). Shown are the estimated rates assuming transcription noise due to promoter ON–OFF switching dynamics driven by transcription factor binding/unbinding (black bars). Also shown are the estimated rates assuming additional noise sources such as accumulation of supercoiling causing promoter locking/unlocking dynamics (grey bars, Table S6). As in Figure 3, all rate constants were estimated from fitting *Eq. 3* and *Eq. 4* to the stochastic simulation data of '*Initially OFF cells*' (light blue) and '*Initially ON'* (light orange) cells, respectively. All fits have R^2 >= 0.6. A total of 500 simulations were performed.

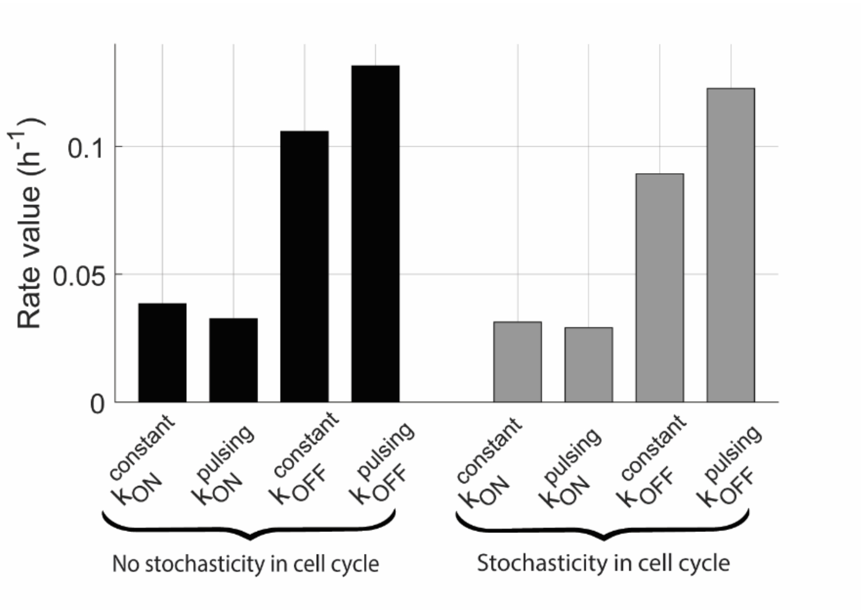

Figure D: Effects of cell cycle stochasticity. Estimated values of the biofilm matrix activation rate assuming constant ($k_{ON}^{constant}$*)* and pulsing 0A~P ($k_{ON}^{pulsing}$*)* and of the matrix deactivation rate assuming constant ($k_{OFF}^{constant}$) and pulsing 0A~P input ($k_{OFF}^{pulsing}$). Shown are the estimated rates assuming deterministic cell cycles with binomially partition of cell volume (black bars). Also shown are the estimated rates assuming cell cycle stochasticity (grey bars). As in Figure 3, all rate constants were estimated from fitting *Eq. 3* and *Eq. 4* to the stochastic simulation data of '*Initially OFF cells*' (light blue) and '*Initially ON'* (light orange) cells, respectively. All fits have R^2 >= 0.69. Over 1000 simulations were performed per condition.

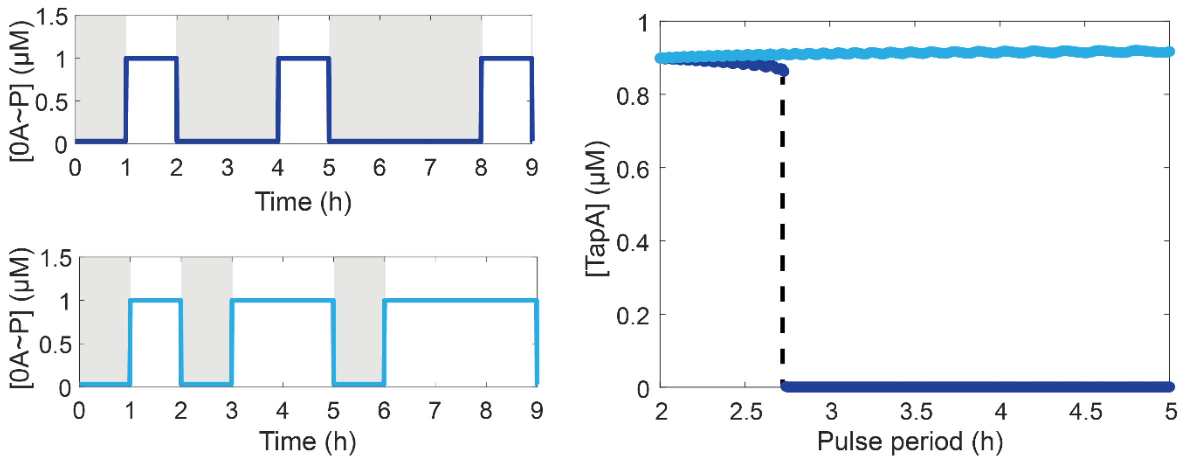

Figure E: Effects of 0A~P OFF and ON time in matrix deactivation. (Upper left) 0A~P square pulse dynamics for increasing OFF time length (grey area) and constant ON time (white area). **(Bottom left)** 0A~P square pulse dynamics for constant OFF time length (grey area) and increasing ON time (white area). The initial pulse given was the same with an ON and OFF time-length equal to 1 h. Square pulse height was kept constant and equal to 1. **(Right)** Dark blue line is the result for the pulse dynamics shown in upper left, i.e., ON time is kept constant while OFF time increases. Light blue line is the result for the pulse dynamics shown in (bottom left), i.e., OFF time is kept constant while ON time increases.

Supplementary References:

1. Narula J, Kuchina A, Zhang F, Fujita M, Süel GM, Igoshin OA. Slowdown of growth controls cellular differentiation. Mol Syst Biol. 2016 May 23;12(5):871.

2. Chen Z, Zarazúa-Osorio B, Srivastava P, Fujita M, Igoshin OA. The slowdown of growth rate controls the single-cell distribution of biofilm matrix production via an SinI-SinR-SlrR network. mSystems. 2023 Apr 27;8(2):e0062222.

3. Eswaramoorthy P, Dinh J, Duan D, Igoshin OA, Fujita M. Single-cell measurement of the levels and distributions of the phosphorelay components in a population of sporulating Bacillus subtilis cells. Microbiology. 2010 Aug 1;156(8):2294–304.

4. Hambraeus G, von Wachenfeldt C, Hederstedt L. Genome-wide survey of mRNA half-lives in Bacillus subtilis identifies extremely stable mRNAs. Mol Genet Genomics. 2003 Aug;269(5):706–14.

5. Sekar V, Hageman JH. Protein turnover and proteolysis during sporulation of Bacillus subtilis. Folia Microbiol (Praha). 1987;32(6):465–80.

6. Chai Y, Kolter R, Losick R. Reversal of an epigenetic switch governing cell chaining in Bacillus subtilis by protein instability. Mol Microbiol. 2010 Oct;78(1):218–29.

7. Palma CSD, Kandavalli V, Bahrudeen MNM, Minoia M, Chauhan V, Dash S, et al. Dissecting the in vivo dynamics of transcription locking due to positive supercoiling buildup. Biochim Biophys Acta Gene Regul Mech. 2020 May;1863(5):194515.

8. Dash S, Palma CSD, Baptista ISC, Almeida BLB, Bahrudeen MNM, Chauhan V, et al. Alteration of DNA supercoiling serves as a trigger of short-term cold shock repressed genes of E. coli. Nucleic Acids Res. 2022 Aug 26;50(15):8512–28.
